# Elevated D-dimer and fibrinogen levels in serum of preoperative bone fracture patients

**DOI:** 10.1186/s40064-016-1817-1

**Published:** 2016-02-24

**Authors:** Chen Liu, Ying Song, Jingzhong Zhao, Qinzhu Xu, Ning Liu, Lei Zhao, Songsong Lu, Hui Wang

**Affiliations:** Department of Clinical Laboratory, Peking University People’s Hospital, Beijing, 100044 China

**Keywords:** Fracture, D-dimer, Fibrinogen, PT INR, APTT

## Abstract

The changes of coagulation parameters in preoperative fracture patients reflect the coagulation status before surgery. We did retrospective assessment of preoperative fracture patients (n = 113) admitted to the hospital between September 2013 and September 2014. The control group were selected from healthy adults (n = 113) with matched age and gender. Platelet, PT INR, APTT, fibrinogen (FIB) and D-dimer values were collected and analyzed. PT INR level was 1.043 ± 0.119, APTT was 31.91 ± 7.56 s, FIB was 320.6 ± 71.8 mg/dl and D-dimer was 1283 ± 1582 ng/ml for the fracture patients. For the control group, PT INR level was 0.9976 ± 0.0602, APTT was 33.22 ± 2.55 s, FIB was 277.3 ± 44.7 mg/dl and D-dimer was 97.53 ± 63.90 ng/ml. Meanwhile, D-dimer levels of different sites of fractures were also measured: Femora 2448 ± 1961 ng/ml; Humerus 792.4 ± 691.2 ng/ml; Ulna/Radius 619.4 ± 843.7 ng/ml; Vertebra 647.7 ± 787.1 ng/ml; Tibia/Fibula 496.3 ± 268.8 ng/ml; Clavicle 260.9 ± 170.9 ng/ml; Ankle 415.4 ± 286.6 ng/ml. To conclude, D-dimer and fibrinogen levels get higher in preoperative fracture patients than controls. Besides, D-dimer levels are significantly different among different locations of fractures, and our data revealed that D-dimer levels of Femora fracture were higher than other sites.

## Background

Fractures, one kind of common disease in Surgery Departments, possesses high incidence in population especially among old people (Broadbent et al. 2003; Feder et al. 2000; Baron et al. 1996). An accurate test of coagulation status is of great value for fracture patients. Regular parameters, including APTT, PT INR, Fibrinogen (FIB) and D-dimer, could reflect the coagulation status of fracture patients, which are prerequisites before surgical operation as well. Prothrombin time (PT) and activated partial thromboplastin time (APTT) are used for analysis of extrinsic and endogenous coagulation pathways (Suchman and Griner 1986). Fibrinogen is a glycoprotein in vertebrates, which could be converted by thrombin into fibrin during blood clot formation (Bini et al. 1989). D-dimer, degradation product of fibrinogen and cross-linked fibrin, could trigger signals for activating hemostasis and fibrinolysis in the blood (Wada et al. 2005). D-dimer levels had been reported to be elevated in patients with cancer, surgery, sepsis, and critical illness (Lippi et al. 2010; Komurcuoglu et al. 2011; Tas et al. 2013a, b; Batschauer et al. 2010; Yamamoto et al. 2012).

Several studies had reported the raise of D-dimer level for fractures (Okamura et al. 2007; Bakhshi et al. 2012; Huang et al. 2013; Owings et al.^2001^; Dindo et al. 2009; Hak 2001; Zhang et al. 2012). However, there are few researches systematically concerning about the change of coagulation parameters. Besides, the change of D-dimer in fractures of different locations is little known. We conducted retrospective study to elucidate the variation of coagulation parameters for fractures. D-dimer levels of different sites of fractures were analyzed in this study as well. Our work will clarify the change of coagulation status of preoperative fracture patients.

## Results

### Comparison of the coagulation parameters between fracture group and the control group

**Fig. 1:**
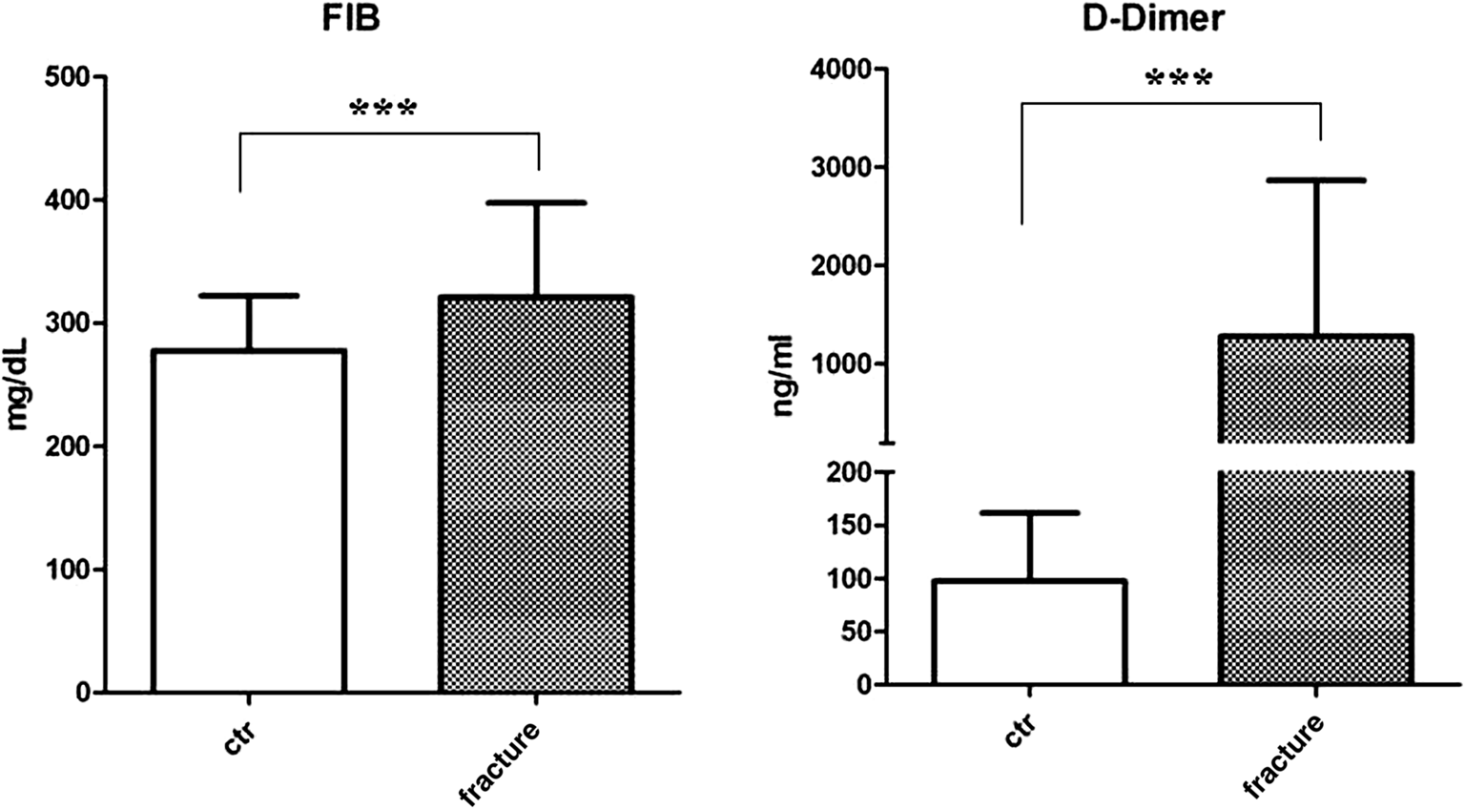
Fibrinogen (FIB) and D-dimer levels in plasma of the fracture patient group and the control group. Data are present as mean ± SD. ***P < 0.001

113 fracture patients with no complications were enrolled, 48 males and 65 females, with mean age of 58.00 ± 18.27. Platelet (PLT), PT INR, APTT, fibrinogen (FIB) and D-dimer of the patients were measured within 1 h after fracture happened before surgery. PT INR level was 1.043 ± 0.119, APTT was 31.91 ± 7.56 s, FIB was 320.6 ± 71.8 mg/dl and D-dimer was 1283 ± 1582 ng/ml. Results of the patients are listed in Table 1. Compared with control, we found that PT INR was higher and APTT was lower in the fracture group, the difference is significant. However, the mean values were close to each other. FIB and D-dimer levels were higher for the fracture group and the difference is significant as well (Fig. 1). According to reference interval of Chinese people, we divided all the individuals into normal (within the reference interval) and abnormal ones (higher or lower than the reference interval). Fisher’s test was used to compare the constitution of fracture group and the control group. We found that PT INR show no significant difference (P > 0.05), but APTT, FIB and D-dimer show P values less than 0.05, suggesting the change of these parameters for fracture patients.

**Table 1:** Comparison of parameters between the fracture group and the control group

	Fracture (n = 113)	Control (n = 113)	P
Male/female	48/65	55/58	0.423
Age (years)	58.00 ± 18.27	60.54 ± 20.89	0.2078
INR	1.043 ± 0.119	0.9976 ± 0.0602	0.0002
<1.2	108	113	0.0579
>1.2	5	0	
APTT (s)	31.91 ± 7.56	33.22 ± 2.55	0.0001
<25.4	5	0	0.0092
25.4–38.4	104	113	
>38.4	4	0	
FIB (mg/dl)	320.6 ± 76.8	277.3 ± 44.7	0.0001
<200	97	113	0.0001
>400	16	0	
D-dimer (ng/ml)	1283 ± 1582	97.53 ± 63.90	0.0001
<250	19	111	0.0001
>250	94	2	
PLT (×10^9^/L)	204.7 ± 63.75	217.6 ± 61.85	0.1227
<125	7	6	0.8624
125–350	103	105	
>350	3	2

### Comparison of D-dimer levels according to different sites of fracture

**Fig. 2:**
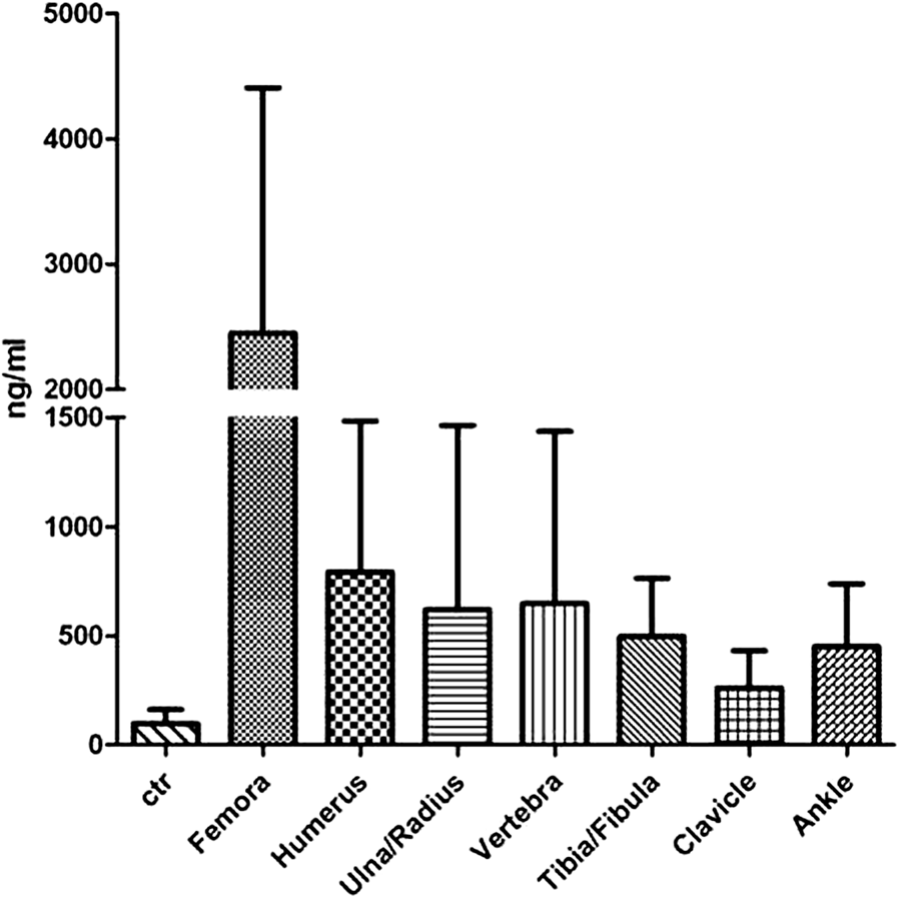
D-dimer levels of fractures at different locations. Data are present as Mean ± SD

Fracture cases analyzed here included fractures of different locations. Then we analyzed D-dimer levels and compared each site of fracture with the control. According to Table 2 and Fig. 2, D-dimer levels of Femora, Humerus, Ulna/Radius, Vertebra, Tibia/Fibula, Clavicle and Ankle fractures were compared with the control. Mann–Whitney test and ANOVA with Dunn’s Multiple Comparison Test were performed. For Mann–Whitney test, we found that all sites of fractures show significant higher D-dimer levels than the control group. Meanwhile, for ANOVA with Dunn’s Multiple Comparison we found only the comparison of Clavicle fracture with the control showed no significant difference (P > 0.05) and all others were significant.

**Table 2:** D-dimer level comparison of different sites of fractures with the control group

	No. of values	Median (ng/ml)	Mean (ng/ml)	Std. deviation (ng/ml)	Std. error (ng/ml)	t test (compared with control)	ANOVA (compared with control)
Femora	43	2232	2448	1961	299	***	***
Humerus	21	525	792.4	691.2	150.8	***	***
Ulna/Radius	14	316	619.4	843.7	225.5	***	**
Vertebra	10	314	647.7	787.1	248.9	***	**
Tibia/Fibula	8	407	496.3	268.8	95.02	***	**
Clavicle	7	234	260.9	170.9	64.6	**	NS
Ankle	10	432.5	451.4	286.6	90.62	***	*
ctr	113	80	97.53	63.9	6.011	N/A	N/A

* P < 0.05; ** P < 0.01; *** P < 0.001; *NS* not significant; *N/A* not applicable

### D-dimer level is higher in Femora fracture patients than other fractures

We could see variation of D-dimer levels according to different locations of fractures in Fig. 2. Fractures of different sites showed quite different levels of D-dimer. We then compared different sites of fractures with each other by ANOVA with Dunn’s Multiple Comparison Test and the results are listed in Table 3. In short, Femora fracture show higher D-dimer levels than that of other locations, and there was no significant difference between other sites of fractures. This cued that Femora fracture produced more D-dimer and the distinguishing expression of D-dimer is interesting, which need to be paid attention to.

**Table 3:** Comparison of D-dimer levels between different fracture of different site

	Femora	Humerus	Ulna/Radius	Vertebra	Tibia/Fibula	Clavicle	Ankle
Femora	N/A	**	***	**	*	***	**
Humerus	**	N/A	NS	NS	NS	NS	NS
Ulna/Radius	***	NS	N/A	NS	NS	NS	NS
Vertebra	**	NS	NS	N/A	NS	NS	NS
Tibia/Fibula	*	NS	NS	NS	N/A	NS	NS
Clavicle	***	NS	NS	NS	NS	N/A	NS
Ankle	**	NS	NS	NS	NS	NS	N/A

* P < 0.05; ** P < 0.01; *** P < 0.001; *NS* not significant; *N/A* not applicable

### Correlation analysis between each coagulation parameter

To elucidate if the change of each parameter is independent, we analyzed the correlation between each coagulation parameter. Spearman test was used for correlation analysis. Correlation coefficient (r) and P values are shown in Table 4. We found that there was a significant correlation between PT and APTT. The correlation between FIB and D-dimer was significant as well. The correlation coefficients (r), however, were just 0.252 and 0.24, which means that the correlations are weak between the parameters.

**Table 4:** Correlation analysis between each coagulation parameter

Parameters	Correlation coefficient (r)	P value	
INR—APTT	0.252	0.0072	**
INR—FIB	0.042	0.6578	NS
INR—D-dimer	0.163	0.0852	NS
APTT—FIB	−0.105	0.2695	NS
APTT—D-dimer	−0.155	0.1021	NS
FIB—D-dimer	0.24	0.0105	*

* P < 0.05; ** P < 0.01; *NS* not significant

## Discussion

This study investigated change of coagulation parameters in preoperative fractures patients, including APTT, PT INR, fibrinogen and D-dimer. As the degradation product of fibrinogen and cross-linked fibrin, D-dimer has been increasingly used particularly in diagnosis of coagulation function disorder diseases (Wada et al. 2005). Several studies demonstrated higher D-dimer values were always observed in patients with cancer, surgery, sepsis, and critical patients (Lippi et al. 2010; Komurcuoglu et al. 2011; Tas et al. 2013a, b; Batschauer et al. 2010; Yamamoto et al. 2012). Accordingly, here we analyzed the role D-dimer plays in fractures, and the changes of the parameters before surgery are worth being carefully considered.

Most articles concerning the relationship between D-dimer and traumas are about the prevention of thrombosis after operation (Okamura et al. 2007; Bakhshi et al. 2012; Huang et al. 2013; Owings et al. 2001; Dindo et al. 2009; Hak 2001). However, there are few literatures systematically discussing coagulation parameter changes in fractures of different locations. Okamura et al. reported that plasma levels of D-dimer and FDP in hip fracture patients were high in perioperative period (Okamura et al. 2007). Bakhshi et al. demonstrated that D-dimer level was high in postoperative lower limb fracture and D-dimer assay was a useful and sensitive test for detecting posttraumatic DVT (Bakhshi et al. 2012). Huang et al. reported that FIB and D-dimer became higher in the hip fracture in elderly patients, before and after surgery (Huang et al. 2013). According to our results, D-dimer levels of different sites of fractures are different, higher in Femora fracture. The mean values of Clavicle and Ankle are much lower. We wonder if fracture located at small skeletons could trigger lower level of D-dimer than big skeletons, and the severity of the trauma may have something to do with the level of D-dimer in plasma as well.

D-dimer is of clinical value when there is a suspicion of deep venous thrombosis, pulmonary embolism or disseminated intravascular coagulation (Stein et al. 2004). Though patients with these complications had been excluded in our research, D-dimer levels were still significantly higher than control. This suggested that pretreated fracture patients were faced with the increased risk of thrombosis. The fracture may lead to microthrombosis which is difficult to be checked as well. The increase of D-dimer in plasma of fracture patients should be paid attention to and related preventive action for thrombosis need to be done. Besides, the risk of bleeding will also rise after thrombosis due to the exhaust of coagulation factors.

Findings of the study present showed the change of FIB and D-dimer, both of them increased in fracture patients. As for APTT and PT INR, we found they underwent a significant change, decreased PT INR and extended APTT, but the mean values were close to the control. We assume that PT and APTT could be affected by fractures, but the changes are limited. From the correlation analysis, we found that the change of the parameters was to some extent independent since the correlation coefficients were small.

## Conclusions

To conclude, coagulation parameters in fractures have been changed. Fibrinogen and D-dimer levels in plasma are elevated, which is of value and could be used for evaluating the coagulation status of preoperative patients. D-dimer levels are diverse according to the location of fracture, higher in Femora fracture, which is of reference meaning in clinical use. Results from our research could help to clarify the change of coagulation status of fracture patients.

## Methods

### Patients

A retrospective study was designed using data obtained from medical records between September 2013 and September 2014 in Clinical Lab of the Hospital with approval from institutional ethical review board of the Hospital. One hundred and thirteen preoperative fracture patients without complications were included in the study, which had been confirmed to be fractures by imaging examination afterwards. One hundred and thirteen healthy adults of matched age and gender were selected in the control group, who were applied to check-up clinic. All members had no active complaint, chronic disease, deep vein thrombosis or other abnormal physical examination. Type of fracture, platelet, PT INR, APTT, FIB and D-dimer levels were recorded.

### Blood sampling and measurement

We collected peripheral venous blood of the patients within 1 h after fracture, using the Vacutainer tubes (Becton–Dickinson) containing 1/10 volume of 0.129 M sodium citrate. Samples were centrifuged at 2200 g for10 min to get the serum of the patients. PT INR (Determined by PT and ISI), APTT, FIB and D-dimer levels were determined on ACL-TOP multiparameter hemostasis analyzer (Instrumentation Laboratory company, Bedford, MA) according to the manufacturer’s instructions. D-dimer HS (Instrumentation Laboratory company, Bedford, MA) is an automated latex enhanced immunoassay, which was used for the quantitative determination of D-dimer in human citrated plasma on the ACLTOP. Platelet (PLT) levels were measured by SYSMEX XE-2100 hematology analyzer.

### Statistics

GraphPad Prime 5.5 was used for calculation of all these significance tests. Mann–Whitney test and ANOVA with Dunn’s Multiple Comparison Test were performed to compare the difference of each coagulation parameter. Fisher’s test was used for comparing categorical variables. Spearman test was used for correlation analysis. A P value less than 0.05 was considered statistically significant,*P < 0.05; **P < 0.01; ***P < 0.001; ns, not significant; N/A, not applicable.

## Abbreviations

APTT: activated partial thromboplastin time
DVT: deep vein thrombosis
FDP: fibrin degradation product
FIB: fibrinogen
INR: international normalized ratio
PLT: platelet
PT: prothrombin time

## References

[CR1] Bakhshi H, Alavi-Moghaddam M, Wu KC, Imami M, Banasiri M (2012). D-dimer as an applicable test for detection of posttraumatic deep vein thrombosis in lower limb fracture. Am J Orthop (Belle Mead NJ).

[CR2] Baron JA, Barrett JA, Karagas MR (1996). The epidemiology of peripheral fractures. Bone.

[CR3] Batschauer APB, Figueiredo CP, Bueno EC, Ribeiro MA, Dusse LMS, Fernandes AP, Gomes KB, Carvalho  MG (2010). D-dimer as a possible prognostic marker of operable hormone receptor-negative breast cancer. Ann Oncol.

[CR4] Bini A, Fenoglio JJ, Mesa-Tejada R, Kudryk B, Kaplan KL (1989). Identification and distribution of fibrinogen, fibrin, and fibrin (ogen) degradation products in atherosclerosis. Use of monoclonal antibodies. Arterioscler Thromb Vasc Biol.

[CR5] Broadbent MR, Quaba O, Hadjucka C, McQueen MM (2003). The epidemiology of multifocal upper limb fractures. Scand J Surg.

[CR6] Dindo D, Breitenstein S, Hahnloser D, Seifert B, Yakarisik S, Asmis LM, Muller MK, Clavien PA (2009). Kinetics of D-dimer after general surgery. Blood Coagul Fibrinolysis.

[CR7] Feder G, Cryer C, Donovan S, Carter Y (2000). Guidelines for the prevention of falls in people over 65. BMJ.

[CR8] Hak DJ (2001). Prevention of venous thromboembolism in trauma and long bone fractures. Curr Opin Pulm Med.

[CR9] Huang W, Xu LY, Shao SY, Yao L, Wang TB (2013). Impact of hip fracture on coagulation function in elderly patients. Beijing Da Xue Xue Bao. Yi Xue Ban = Journal of Peking University. Health Sciences.

[CR10] Komurcuoglu B, Ulusoy S, Gayaf M, Guler A, Ozden E (2011). Prognostic value of plasma D-dimer levels in lung carcinoma. Tumori.

[CR11] Lippi G, Cervellin G, Franchini M, Favaloro EJ (2010). Biochemical markers for the diagnosis of venous thromboembolism: the past, present and future. J Thromb Thrombolysis.

[CR12] Okamura K, Nakagawa I, Hidaka S, Okada Y, Kubo T, Kato T (2007). Perioperative changes of the coagulation markers in patients undergoing hip fracture surgery. Masui Jpn J Anesthesiol.

[CR13] Owings JT, Gosselin RC, Anderson JT, Battistella FD, Bagley M, Larkin EC (2001). Practical utility of the D-dimer assay for excluding thromboembolism in severely injured trauma patients. J Trauma Acute Care Surg.

[CR14] Stein PD, Hull RD, Patel KC, Olson RE, Ghali WA, Brant R, Biel RK, Bharadia V, Kalra NK (2004). D-dimer for the exclusion of acute venous thrombosis and pulmonary embolism: a systematic review. Ann Intern Med.

[CR15] Suchman AL, Griner PF (1986). Diagnostic decision: diagnostic uses of the activated partial thromboplastin time and prothrombin time. Ann Intern Med.

[CR16] Tas F, Kilic L, Bilgin E, Keskin S, Sen F, Ciftci R, Yildiz I, Yasasever V (2013). Clinical and prognostic significance of coagulation assays in advanced epithelial ovarian cancer. Int J Gynecol Cancer.

[CR17] Tas F, Kilic L, Serilmez M, Keskin S, Sen F, Duranyildiz D (2013). Clinical and prognostic significance of coagulation assays in lung cancer. Respir Med.

[CR18] Wada H, Sase T, Yamaguchi M (2005). Hypercoagulant states in malignant lymphoma. Exp Oncol.

[CR19] Yamamoto M, Yoshinaga K, Matsuyama A, Iwasa T, Osoegawa A, Tsujita E, Yamashita Y, Tsutsui S, Ishida T (2012). Plasma D-dimer level as a mortality predictor in patients with advanced or recurrent colorectal cancer. Oncology.

[CR20] Zhang LD, Liu HB, Li YN, Ma HM, Liu YB, Wang MY (2012). Correlation analysis between plasma D-dimer levels and orthopedic trauma severity. Chin Med J.

